# Neurophysiological signatures of mild traumatic brain injury in the acute and subacute phase

**DOI:** 10.1007/s10072-024-07364-4

**Published:** 2024-02-17

**Authors:** Valentina Barone, Myrthe E. de Koning, Harm J. van der Horn, Joukje van der Naalt, Carin J. Eertman-Meyer, Michel J. A. M. van Putten

**Affiliations:** 1 Clinical Neurophysiology (CNPH), TechMedCenter, University of Twente, Drienerlolaan 5, 7500 AE Enschede, The Netherlands; 2 Department of Clinical Neurophysiology, Medisch Spectrum Twente, Koningsplein 1, 7512 KZ Enschede, The Netherlands; 3 Department of Neurology, University of Groningen, UniversityMedicalCenterGroningen, Hanzeplein 1, 9713 GZ Groningen, The Netherlands

**Keywords:** Visual attention, Mild traumatic brain injury, EEG, ERP, Eye tracking

## Abstract

**Background:**

Mild traumatic brain injury (mTBI) affects 48 million people annually, with up to 30% experiencing long-term complaints such as fatigue, blurred vision, and poor concentration. Assessing neurophysiological features related to visual attention and outcome measures aids in understanding clinical symptoms and prognostication.

**Methods:**

We recorded EEG and eye movements in mTBI patients during a computerized task performed in the acute (< 24 h, TBI-A) and subacute phase (4–6 weeks thereafter). We estimated the posterior dominant rhythm, reaction times (RTs), fixation duration, and event-related potentials (ERPs). Clinical outcome measures were assessed using the Head Injury Symptom Checklist (HISC) and the Extended Glasgow Outcome Scale (GOSE) at 6 months post-injury. Similar analyses were performed in an age-matched control group (measured once). Linear mixed effect modeling was used to examine group differences and temporal changes within the mTBI group.

**Results:**

Twenty-nine patients were included in the acute phase, 30 in the subacute phase, and 19 controls. RTs and fixation duration were longer in mTBI patients compared to controls (*p* < 0.05), but not between TBI-A and TBI-S (*p* < 0.05). The frequency of the posterior dominant rhythm was significantly slower in TBI-A (0.6 Hz, *p* < 0.05) than TBI-S. ERP mean amplitude was significantly lower in mTBI patients than in controls. Neurophysiological features did not significantly relate to clinical outcome measures.

**Conclusion:**

mTBI patients demonstrate impaired processing speed and stimulus evaluation compared to controls, persisting up to 6 weeks after injury. Neurophysiological features in mTBI can assist in determining the extent and temporal progression of recovery.

**Graphical abstract:**

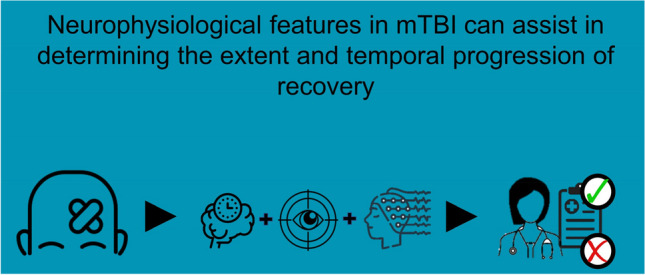

**Supplementary Information:**

The online version contains supplementary material available at 10.1007/s10072-024-07364-4.

## Introduction

Mild traumatic brain injury (mTBI) affects approximately 48 million people per year worldwide [1, 2]. Post-traumatic complaints include headache, forgetfulness, fatigue, and a reduced attention span [3, 4]. While most patients recover within 3 months after injury, about 15–30% report post-traumatic complaints beyond this period [5, 6], often related to visuomotor activities and visual attention [7–11].

Assessment of visuomotor activities and visual attention both in the acute (i.e., within 24 h after trauma) and subacute (4–6 weeks after trauma) phases of mTBI, together with outcome measures linked to complaints, can be helpful for a more detailed evaluation of the severity of the condition and outcome prediction [7–9]. Several neurophysiological techniques have been explored for such assessment, including reaction times, electroencephalography (EEG), measurement of event-related potentials (ERPs), and evaluation of oculomotor functions [6, 12–16].

The latency between the appearance of a visual stimulus and the production of a motor response, i.e., visual reaction time (RT), is usually longer in patients with mTBI, both in the acute and subacute phases [7, 15]. RT can be divided into additional subcomponents, employing an eye tracker (ET). Analysis of these subcomponents helps to define which degree of stimuli processing, evaluation, or motor production is significantly impaired. For example, it was shown that processing speed—i.e., latency between the moment when the eyes reach the visual target and a behavioral response, which is related to stimulus recognition, decision-making, and motor activity—was affected the most after mild and severe TBI [12]. The posterior dominant EEG rhythm (PDR) is usually slower directly after mTBI [17], with a gradual increase of 1–2 Hz in the alpha band up to weeks after the trauma [17]. Late components of ERPs related to visual attention are often affected in mTBI patients [15, 18]. In particular, the peak latency of the P300 was significantly prolonged in early mTBI compared to controls [18], suggesting a delayed evaluation and categorization of stimuli [18] and a slower neural conduction [19]. A smaller P300 mean amplitude was also observed in patients with mTBI, expressing a poor performance of attentive and cognitive resources [19, 20]. Eye movements can also express disturbed visuoattentional performance after brain injury since they share common neuronal circuitry [21, 22], and it has been shown that oculomotor functions can be impaired in patients with mTBI [11].

Most studies in mTBI used single neurophysiological techniques, which may not always reflect the heterogeneity of complaints and presumed disturbances in brain function. Here, we investigated a combination of neurophysiological biomarkers of visuospatial attention in patients with mTBI, both in the acute stage and 4–6 weeks after the trauma and compared these results with controls. We recorded RTs, EEG, and eye movements with a fast and portable task that allows simultaneous measurement of all three metrics [23]. Furthermore, we assessed the clinical outcome of mTBI 6 months after the injury, quantified with the Head Injury Symptom Checklist (HISC) [3, 24] and the Extended Glasgow Outcome Scale (GOSE) [25]. We hypothesized that both EEG and ET may provide sensitive features to differentiate patients with mTBI from controls. In addition, we related these features to long-term clinical outcomes. In this exploratory research, we explore potential indicators for mTBI diagnosis and the factors influencing patient recovery.

## Methods

### Subjects

We included non-epileptic, adult participants, without cognitive deficits: 19 healthy controls (mean age = 42 ± 15, 5 females), 29 mTBI patients in the acute phase (TBI-A, mean age ± 46 ± 20, 12 females), and 30 mTBI patients in the subacute phase (TBI-S, mean age = 51 ± 18, 14 females). Twenty-eight mTBI patients were included both in the acute and subacute phases. mTBI patients were defined by a Glasgow Coma Scale score between 13 and 15 and transient loss of consciousness < 30 min and/or post-traumatic amnesia < 24 h [26]. Patients were admitted to the emergency department of the Medisch Spectrum Twente, Enschede, the Netherlands. Our study is part of the AIM-TBI study (Netherlands Trial Register number NL8484), a Dutch, multicenter study involving the University Medical Center Groningen and the Medisch Spectrum Twente. Patients’ inclusion complied with the Declaration of Helsinki and was approved by the Medical Ethical Committee of the University Medical Center Groningen (METc 2018/681). All participants provided written informed consent, according to the approved research protocol.

### Task and procedure

To measure visual attention we employed a custom-built computerized choice reaction time task (CRT), synchronized with a 19-channel EEG and a screen-based eye tracker, previously described [23]. Measurements for TBI patients were performed at the Medisch Spectrum Twente, in an office environment for controls. For TBI patients only, resting EEG with eyes closed (EC) and eyes open (EO) was assessed at the beginning of the measurement for 15 min. Both TBI patients and controls performed the CRT task, with a duration of approximately 15 min. During task performance, participants were seated in front of a laptop screen and instructed to direct their gaze towards the visual stimuli appearing sequentially on the screen—i.e., (1) a fixation cross, (2) a white dot, and (3) the face of a monkey with two hands covering the eyes (target stimulus) (cf. Figure S1 of Supporting Information). When either the left or the right hand of the target stimulus was colored in black, participants were instructed to press the corresponding button on a game controller as fast as possible. A total of 112 trials were collected for each participant.

### Eye tracking and reaction times

After performing an automated five-point calibration procedure of our screen-based ET (Tobii Pro Nano, Tobii Technology, Danderyd, Sweden) at the beginning of the CRT task, we obtained the *x*- and *y*-coordinates of both eyes for each time stamp during the task (sampling frequency = 60 Hz, accuracy = 0.3° in optimal conditions, precision = 0.10° RMS in optimal conditions). Eye movement analyses were performed with custom scripts in Python 3.8.3. For more details, see [23]. We defined fixation duration as the time between the first and last samples within the area of interest (AOI) around the target stimulus. Total reaction time (RT) was determined as the time between the target appearance and a correct button press. Using ET, we extrapolated three RT subcomponents: (i) saccadic latency (SL), i.e., sampling time between target appearance and last sample within the AOI around the fixation cross; (ii) visual reaction time (VRT), i.e., sampling time from the first sample outside the AOI of the fixation cross to the first sample within the target AOI; and (iii) processing speed (PS), i.e., the sum of SL and VRT subtracted from the total RT.

### EEG

The EEG was recorded at 512 Hz with a portable Neurocenter EEG system (SAGA + 32, Twente Medical Systems International (TMSi) B.V., the Netherlands), using Ag/AgCl electrodes (10–20 system). Electrode impedance was kept below 20 kΩ. EEG data analysis was performed with Neurocenter, MATLAB (version R2018b), and the freely available EEGLAB (version 2021.0).

#### EEG features of resting EEG

Resting EEG traces during eyes closed (EC) and eyes open (EO) (available for TBI patients only) were filtered offline using a Hamming windowed FIR band-pass filter between 1 and 30 Hz. Artifacts were manually selected and removed from the EEG. For each patient, we estimated the power spectral density (PSD) for every 10-s window of EO and EC EEG, respectively (MATLAB pwelch, with a window length of 4 s and 50% overlap). We then extracted the posterior dominant rhythm (PDR) averaging each PSD obtained in the alpha-band range (8–13 Hz) for parieto-occipital channels. PDR was then normalized by dividing each PDR value by the average PDR. From the PDR of each patient during EC, we determined the peak frequency in the alpha-band range.

#### Event-related potentials

EEGs during CRT task of patients and controls were filtered offline using a Hamming windowed FIR band-pass filter between 1 and 30 Hz. Data were epoched 200 ms prior and 1200 ms after the trigger events (i.e., stimulus appearance), with baseline correction. To discard noisy trials, we applied both a manual and automatic artifact rejection, marking epochs containing peak-to-peak activity greater than 100 µV, within a moving window (interval—200 to 1200 ms, width 200 ms; steps 50 ms). Trial exclusion rate varied between 0 and 75% (20–30 artifact-free trials are enough to estimate P300 and late ERP components [27, 28]). We determined P300 and late ERP components for visual attention allocation (i.e., between 250 and 750 ms from the start of the stimulus) from channels Pz, P3, and P4 [29, 30] averaging the EEG trials and computing the grand average. For each subject, we extrapolated mean amplitude and peak latency for channels Pz, P3, and P4 and averaged the results.

### Clinical outcome measures

Twenty-seven TBI-A and 28 TBI-S patients (26 overlap) completed the HISC and GOSE questionnaires 6 months after injury. The HISC includes 19 frequently reported complaints [3, 24]. Final HISC scores were calculated by comparing scores before and after the trauma: a score of 1 was assigned for every complaint increase after trauma, and 0 if no difference or a complaint decrease was reported. HISC sum scores ranged between 0 and 17. The GOSE is an 8-point ordinal scale consisting of a hierarchy of discrete categories, from death to complete recovery [31]. GOSE was dichotomized between 0 (complete recovery, GOSE = 8) and 1 (incomplete recovery, GOSE < 8).

### Statistics

Our study is a prospective longitudinal cohort study, in which controls underwent one measurement only, while most of the mTBI patients (*N* = 21) underwent the measurement twice. Since our TBI-A and TBI-S groups only partially overlap, we consider them as two separate groups, accounting for the differences in their composition. We divided our dependent variables representing neurophysiological features into three categories: resting EEG (i.e., PDR peak frequency); ERP (i.e., mean amplitude and peak latency); and eye tracking (ET) (i.e., RT, SL, VRT, PS, and fixation duration). We verified the normality of our dependent variables by visualizing the data distribution per group (histogram), inspecting Q-Q plots for normality, and applying the Shapiro–Wilk test. Visually, our data appeared normally distributed (see section S9 of Supporting Information for an example) for all our groups, but Shapiro–Wilk tests indicated only two normally distributed variables (*p* > 0.05), namely, ERP mean amplitude and ET fixation duration. Our data deviated only slightly from normality. Therefore, we considered them further as normally distributed. We defined a linear mixed-effect model to analyze each of our ERP and ET variables as

1 $$y\sim 1+mTBI+mTBI :visit+(1|Subject)$$

where the control group is the reference of our mTBI term, and the mTBI:visit term captures longitudinal effects for the mTBI group.

We selected a linear mixed-effect model due to the model’s capability to accommodate data with partially repeated measures, as seen in our TBI-A and TBI-S groups. Furthermore, linear mixed models are relatively robust to deviations from normality. We determined the normality of the residuals of our models (seven in total). The residuals followed a normal distribution visually (Q-Q plots, histogram; see section S10 of the Supporting Information for an example), but did not pass the Shapiro–Wilk test. Taking this into consideration, we proceeded with parametric statistics. We applied type III ANOVA to assess the significance of the fixed-effect coefficients of our linear models collectively. We corrected *p*-values (Pfdr) for multiple tests for ERP (i.e., two tests) and ET (i.e., five tests) variables, separately. We applied false discovery rate correction, using Benjamini–Hochberg correction (*Q* = 5%).

We then conducted pairwise comparisons to examine differences between the three groups for ET and ERP variables. Pairwise comparisons allowed us to assess the significance of specific differences between groups. To double-check the robustness of our results, we conducted a pairwise comparison of our ERP and ET variables using non-parametric statistics, too (cf. section S8 of Supporting Information).

For our EEG feature (PDR peak frequency), we compared the mean ranks of resting EEG features across TBI-A and TBI-S only, and therefore, we used a Mann–Whitney *U* test. We applied Spearman’s correlation to compare the relationship between outcomes, RTs, and EEG variables. Results were considered statistically significant when *p* < 0.05. Statistical analysis was performed using the module *statistics* in Python 3.8 for pairwise comparison of resting EEG and the packages *lme4*, *emmeans*, and *car* in R 4.3.1 [32] for ERP and ET variables.

## Results

We included 31 patients with mTBI; of these, two did not perform our measurement in the acute phase and one patient did not complete the follow-up 4–6 weeks after trauma. After excluding noisy data, we performed our analyses of resting EEGs on 27 TBI-A and 26 TBI-S and our ERP analyses on 19 controls, 19 TBI-A, and 23 TBI-S patients. Of these, 21 patients completed the measurement both in the acute and subacute phases. The overall demographics of patients and controls are summarized in Table 1. Causes of mTBI are shown in Table 1. Detailed demographics of controls and patients, including medications, are summarized in Tables S1 and S2 of the Supporting Information.

**Table 1:** Demographic characteristics of patients and controls and causes of mTBI. Abnormal CT findings included minor traumatic subarachnoid and subdural hemorrhage or minor hemorrhagic concussion. For details on pre-injury medication type, see Table S1 of the Supporting Information. *SD* standard deviation, *y* years

	Controls (*N* = 19)	TBI-A (*N* = 29)	TBI-A (*N* = 29)	*p*-value
Mean age (y), mean, SD	42 ± 15	46 ± 20	51 ± 18	0.5^a^
Females (%)	26	41	47	0.4^b^
Abnormal CT scan (%)	-	34	40	0.9^b^
Medication (%)	-	28	37	0.9^b^
**Causes of mTBI**				
Bike*/*scooter accident (%)	-	70	68	0.8^b^
Motorcycle accident (%)	-	8	7	0.8^b^
Sport (%)	-	0	3	0.8^b^
Fall (%)	-	22	21	0.8^b^

^a^Kruskal-Wallis test

^b^Pearson’s *χ*^2^ test

### Eye tracking variables: Reaction times and fixation duration

**Fig. 1:**
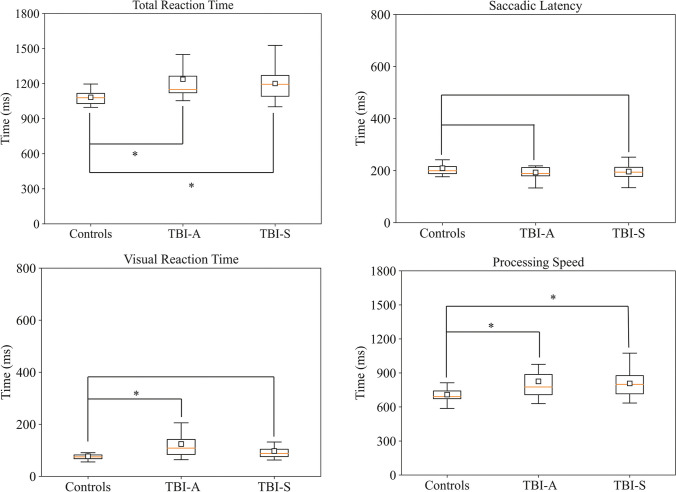
Reaction times. Total RT and RT subcomponents from the CRT task. Total RT is higher for TBI-A compared to TBI-S and controls. Visual reaction time (VRT) is higher in TBI-A, as well (mean response change from controls to mTBI is 45 ms). Processing speed (PS) is comparable between TBI-A and TBI-S (804 vs 806 ms, respectively), but was notably lower in controls (714 ms). Fixed-effect coefficients for the mTBI term and post hoc analysis between controls and mTBI singularly are significant (*p* < 0*.*05) for total RT, VRT (TBI-A, only), and PS, but not for saccadic latency (SL)

ET variables were affected by mTBI. Linear mixed model analysis showed a significant effect of mTBI on RT (test statistic with chisq = 9.3, corrected *p*-value Pfdr = 0.004, mTBI > controls). No significant mTBI:visit term was found (Pfdr = 0.7), indicating no significant change over time within the mTBI group. Post hoc analyses showed a significantly higher RT in mTBI at both the acute (*p* = 0.01) and subacute (*p* = 0.02) timepoints relative to HC. We found a significant influence of mTBI on VRT, as well (chisq = 13.3, Pfdr = 0.01, mTBI > controls), but no significant mTBI:visit term was found (Pfdr = 0.1). Post hoc comparison showed a significant increase in VRT in TBI-A (*p* = 0.02) but not for TBI-S (*p* = 0.09). Processing speed is also affected by mTBI (chisqr = 5, Pfdr = 0.03, mTBI > controls), but no significance was found for mTBI:visit (Pfdr = 0.9). Significantly higher PS was found in TBI-A and TBI-S compared to controls (controls vs TBI-A *p* = 0.04; controls vs TBI-S *p* = 0.04). No significant effect of mTBI was found on saccadic latency (chisqr = 2, Pfdr = 0.1, mTBI > controls), and this was confirmed by post hoc analysis (controls vs TBI-A *p* = 0.2; controls vs TBI-S *p* = 0.2). In summary, RT, VRT, and PS were significantly higher for TBI-A patients compared to controls. RT and PS were higher in TBI-S with respect to controls. SL did not present any significant difference between mTBI and controls (Fig. 1).

In section S3 of the Supporting Information, we present RT subcomponents with the corresponding values and we discuss the influence of age on RTs.

**Fig. 2:**
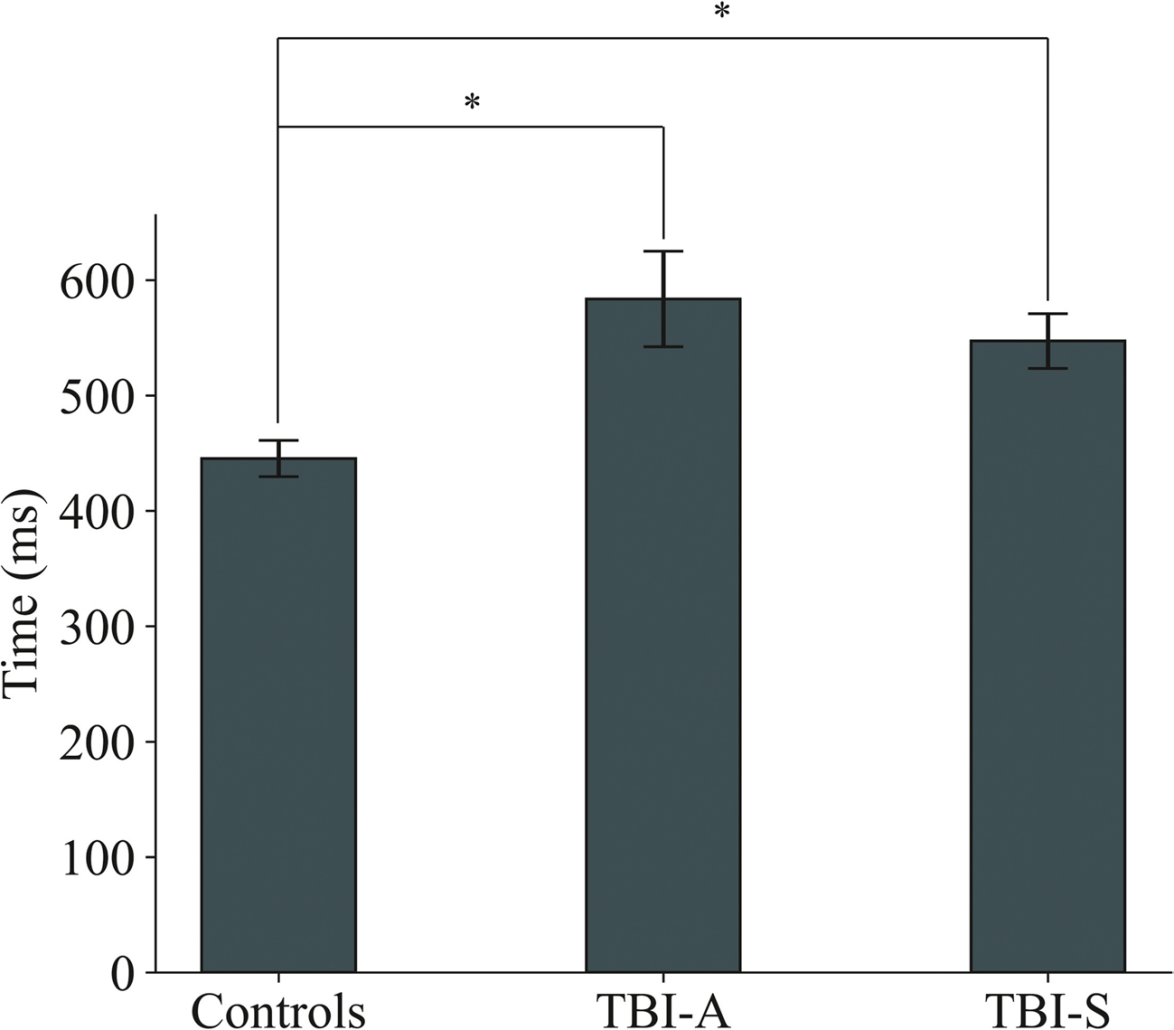
Fixation duration. Fixation duration, defined as the sampling time of the eyes within the area of interest around the target stimulus during the CRT task. Fixation duration is significantly impacted by mTBI. Post hoc analysis determined discrepancies between controls and TBI-A or TBI-S singularly. The mean response change from controls to mTBI is 139 ms

We found a significant effect of mTBI on fixation duration (Fig. 2) (chisq = 10.1, Pfdr = 0.002; mTBI > controls), but no difference was found between TBI-A and TBI-S (mTBI:visit Pfdr = 0.7). Post hoc analysis presented significant differences between controls and mTBI, both acute and subacute (controls vs TBI-A *p* = 0.006; controls vs TBI-S *p* = 0.02).

These results were replicated using non-parametric statistics in section S8 of the Supporting Information, except for VRT, which exhibited significant differences between controls and TBI-S, as well.

### Resting EEG features

**Fig. 3:**
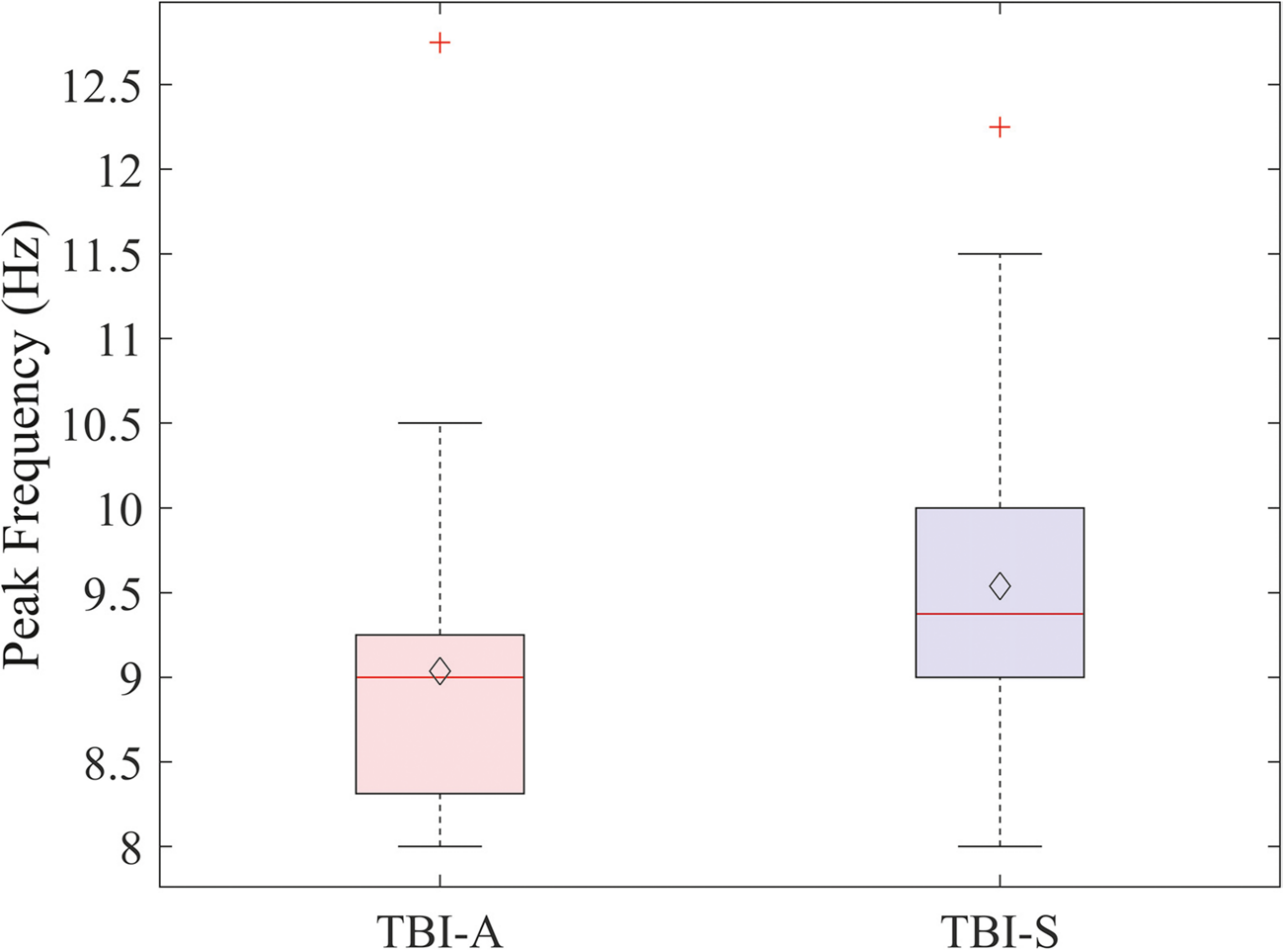
PDR peak frequency. Peak frequency of parieto-occipital channels in the alpha band during resting EEG with eyes closed. A mean difference of 0.6 Hz is found between TBI-A and TBI-S (*p* = 0.02). Red line: median; diamond: mean

During resting EEG with EC, patients showed a significantly (*p* = 0.02) lower PDR peak frequency in the alpha range (8–13 Hz) for parieto-occipital channels in the acute stage (PDR of TBI-A = 9 Hz) compared to the subacute stage (PDR of TBI-S = 9.6 Hz) (Fig. 3).

### Event-related potentials

**Fig. 4:**
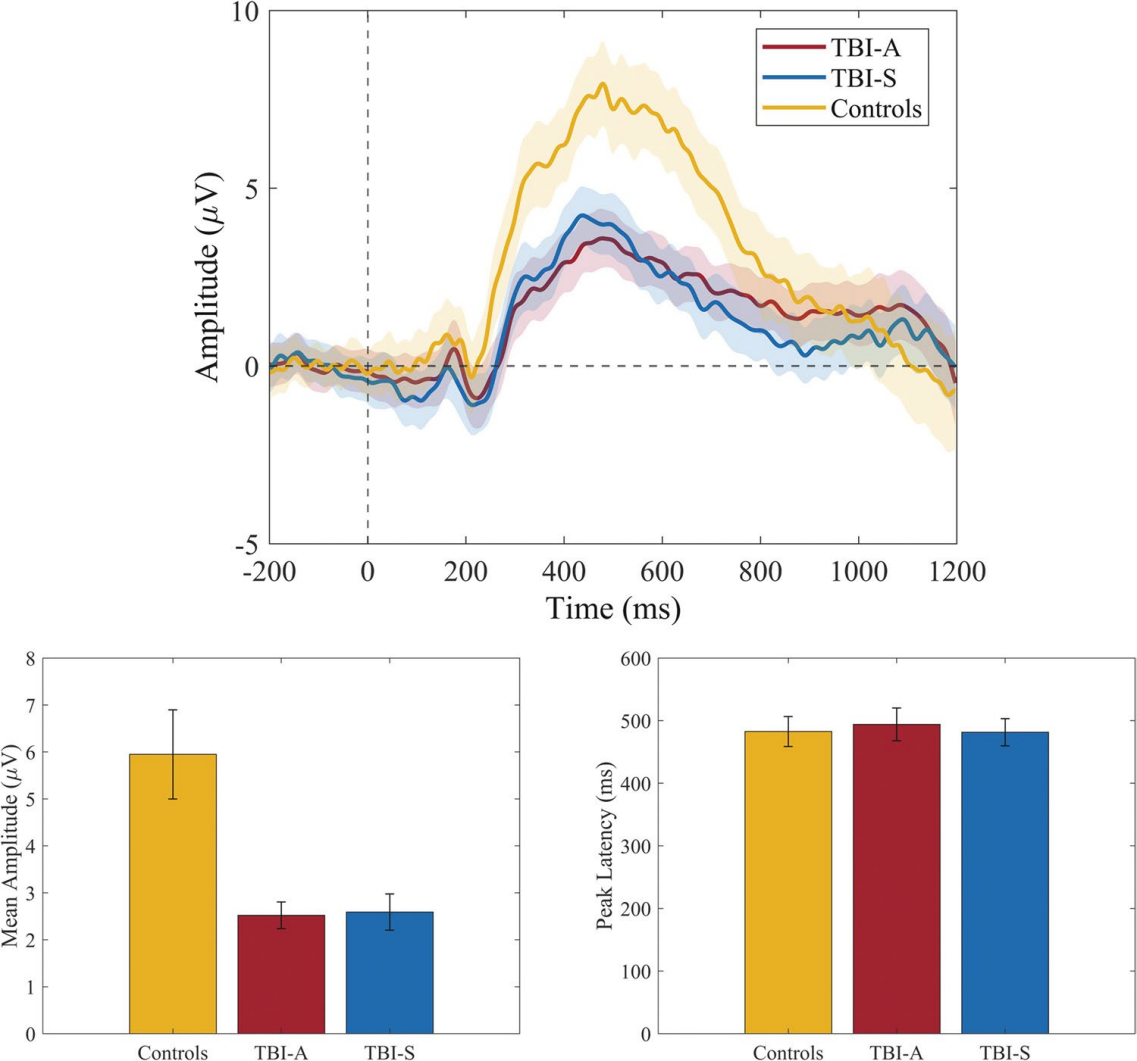
Event-related potentials. Top: grand average ERP for channel P3, Pz, and P4 of the three groups. The colored area around the averaged signal represents the standard error of the mean for each time point. Bottom left: the mean amplitude between 250 and 750 ms is significantly different across groups. The mean difference (MD) between controls and both TBI groups is 3.4 µV. No significant difference is found between TBI-A and TBI-S. Bottom right: peak latency between 250 and 750 ms. No significant difference across groups is found

In Fig. 4, we present the grand average ERP of channels Pz, P3, and P4 for the three groups. Linear mixed model analysis showed a significant effect of mTBI on mean amplitude at 250–750 ms (chisq = 18.9, Pfdr = 2.8 × 10^−5^, mTBI > controls). No significant mTBI:visit term was found (Pfdr = 0.3), indicating no significant change over time within the mTBI group. Post hoc analyses showed a significantly higher mean amplitude in mTBI at both the acute (*p* = 2.1 × 10^−4^) and subacute (*p* = 6.6 × 10^−4^) timepoints relative to controls. mTBI did not affect peak latencies significantly (chisq = 0.2, Pfdr = 0.6, mTBI > controls) and mTBI:visit was not significant, too (Pfdr = 0.5). No differences in peak latency of mTBI were found both for TBI-A (*p* = 0.9) and TBI-S (*p* = 0.9).

These results were verified using non-parametric statistics in section S8 of the Supporting Information.

In section S4 of the Supporting Information, we discuss the influence of age on ERP results.

We repeated our analyses of neurophysiological features on patients with double measurements, only, to control for possible physiological inter-subject differences. In section S7 of the Supporting Information, we demonstrate that all the significant differences we found are preserved.

### Relation of neurophysiological metrics with clinical outcome measures

**Fig. 5:**
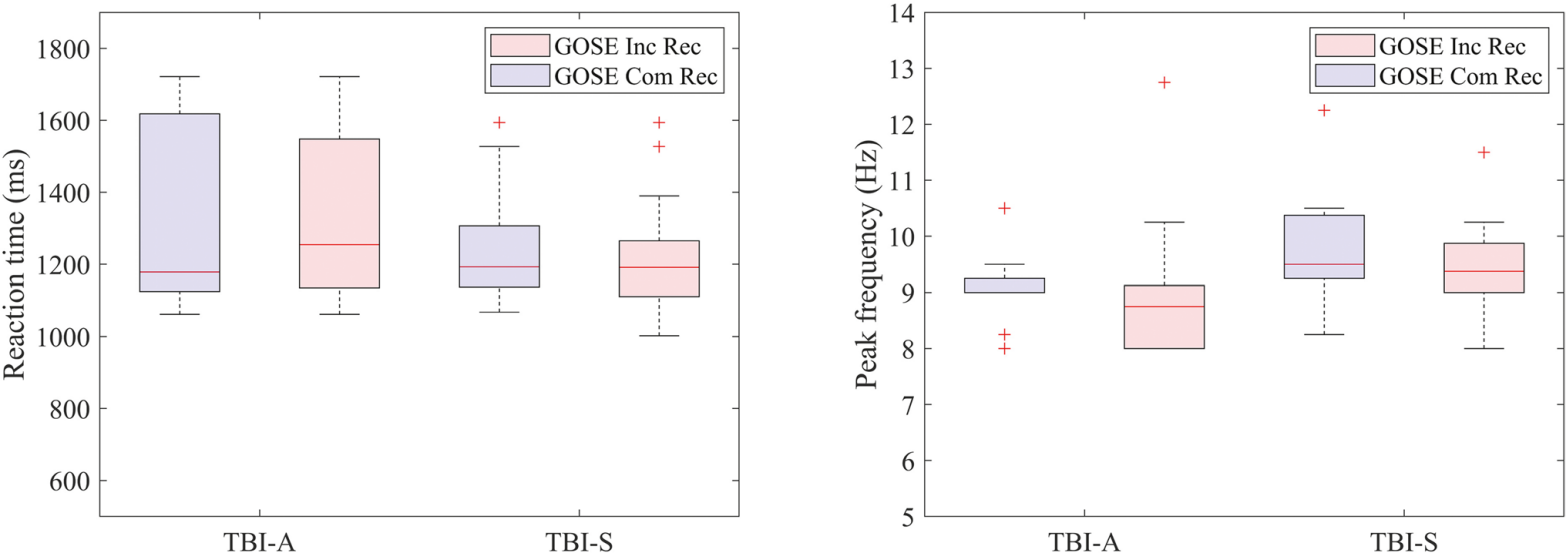
GOSE outcome and neurophysiological metrics. Left: association between GOSE outcomes and total RT for TBI-A and TBI-S. Both mTBI patients with incomplete (pink) and complete (purple) recovery in the acute and subacute phases show a similar median RT (median Inc Rec TBI-A and TBI-S = 1.2 s; median Com Rec TBI-A = 1.3 s, TBI-S = 1.2 s; Spearman’s correlation RT vs GOSE: TBI-A: *p* = 0.1; TBI-S: *p* = 0.4). Right: association between GOSE outcomes and peak frequency in the posterior, alpha band during resting EEG with eye closed for TBI-A and TBI-S. Both groups show a similar median peak frequency with complete (purple) and incomplete (pink) recovery of GOSE scores (median Inc Rec TBI-A = 9.3 Hz, TBI-S = 9.5 Hz; median Com Rec TBI-A = 9.8 Hz, TBI-S = 9.4 Hz; Spearman’s correlation peak frequency vs GOSE: TBI-A: *p* = 0.2; TBI-S: *p* = 0.6). Inc Rec, incomplete Recovery; Com Rec, complete recovery

HISC scores ranged between 0 and 17 and GOSE scores ranged between 6 and 8. Complete recovery in the GOSE (score = 8) was significantly correlated with fewer complaints in the HISC at 6 months post-injury (cf. section S6 of the Supporting Information). Forty-four percent of mTBI patients expressed incomplete recovery 6 months post-injury, i.e., upper moderate disabilities (19%) or lower good recovery (26%). Forty-four percent of mTBI patients described more than three persistent complaints 6 months after injury. Employing Spearman’s correlation, HISC scores did not show any significant correlation (cf. Fig. S4 of the Supporting Information) with total RT and PDR peak frequency in the alpha band. Similarly, we found no significant association between our neurophysiological features and dichotomized GOSE scores (cf. Fig. 5).

## Discussion

In this work, we aimed to assess neurophysiological signatures related to visual attention in patients with mTBI and controls. Further, we determined the association of neurophysiological features with clinical outcomes of mTBI patients. Reaction times, fixation duration, frequency of the PDR, and ERP mean amplitude were significantly different between controls and mTBI patients in both the acute and subacute phases. The only neurophysiological signature changing over time was the peak frequency of the PDR in the posterior channels, which increased from acute to subacute mTBI. Neurophysiological features were however not associated with long-term measures of clinical outcomes and complaints 6 months after injury.

### Reaction times

Patients with mTBI show slower responses for total reaction time, visual reaction time, and processing speed. Our results corroborate previous studies, revealing a longer RT after mTBI [7, 15]. We extended these findings, investigating RT subcomponents, too, with the use of the eye tracker. Our findings indicate that processing speed is impaired after mTBI, stressing a peculiar delay in stimulus evaluation and motor response. This was reported in mild-to-severe TBI patients by Lange and colleagues [12], as well. Here, we established that a longer PS is present both within 24 h and 4–6 weeks after mTBI. Visual reaction time was longer too, with TBI-A showing the longest VRT. A delayed VRT possibly indicates an impairment of oculomotor functions after mTBI. Saccadic latency is comparable among the three groups.

Fixation duration was significantly longer in patients with TBI-A (cf. Figure 2). Fixation duration, i.e., the sampling time spent on the target stimulus, overlaps partially with PS, and they both reveal insights about the speed of stimulus evaluation. Overall, consistently with previous studies [12, 33], RTs, and oculomotor functions seem to be impaired both immediately and up to 6 weeks after the trauma.

### EEG features

In the acute phase, patients with mTBI showed a significant slowing of the posterior alpha peak frequency of 0.6 Hz (PDR of TBI-A = 9 Hz, TBI-S = 9.6 Hz) (cf. Figure 3). The longitudinal increase in the frequency of the PDR may reflect recovery of neuronal functioning, due to functional compensatory responses during early phases of mTBI [34]. EEG alpha power consistently estimates attention changes in the brain [35–38]. A decrease in alpha power, especially in parieto-occipital regions, usually indicates more pronounced employment of attention [36, 37], specifically towards visual external stimuli [38]. Previous literature has shown that a generalized or focal slowing of the EEG is present for weeks after mild, moderate, and severe TBI [14, 39, 40]. In particular, the alpha rhythm is more prominent compared to controls [17]. The posterior alpha frequency is slower by an average of 0.7 Hz directly after trauma, and it returns to baseline over weeks to a few months after injury [17]. In our study, mTBI patients present a slower peak frequency of the PDR a few hours after the trauma compared to 4–6 weeks after mTBI. This may account for a more pronounced impairment of cognitive functioning and attention. Of the chosen variables for assessing visual attention after mTBI (i.e., PDR, ERP, ET), only the PDR’s peak frequency appears to capture the potential dynamic difference between acute and subacute mTBI.

Although the PDR of mTBI patients fell within the established normal range of 8–13 Hz [41, 42], our findings indicate that in the acute phase of mTBI, the PDR exhibited a slower frequency compared to the subacute phase. Given that the PDR values are already within the normal range, there is no necessity for a control group, as the normal values are well-established.

### Event-related potentials

Late ERP components (cf. Figure 4) show a significantly smaller mean amplitude in mTBI patients compared to controls, confirming results from previous studies [20]. A smaller mean amplitude of late ERP components may reflect a disruption of neuronal functioning underlying stimulus evaluation processing. In contrast to a previous study [18], peak latency did not differ between mTBI patients and controls. In ERP studies, peak latency is often subject to noisy data; therefore, mean amplitude may be a more reliable metric for the quantification of ERPs [43].

Age influences ERPs [19, 44]; therefore, we repeated our ERP analysis excluding patients older than 68 years old, in order to obtain a comparable mean age for our three groups (controls 42, TBI-A 41, and TBI-S 46). In section S4 of the Supporting Information, we show that age does not alter our ERP results.

Although numerous studies have explored the application of EEG in the assessment of TBI, recent findings suggest that quantitative EEG may not offer significant utility within the scope of mTBI [45]. In particular, there is a call for studies that incorporate multiple EEG measures representing diverse neurophysiological aspects. In this study, we addressed this need and enhanced it by incorporating additional neurophysiological variables.

### Outcome measures

Approximately 48% of our mTBI patients (*N* = 13) experienced persistent complaints up to 6 months after injury, as defined by three or more complaints from the HISC scores. This roughly corresponds to what was reported in mTBI before [5, 6]. Contrary to our hypothesis, our neurophysiological features did not show any significant relationship with complaints reported in the HISC (cf. section S5 of the Supporting Information) and functional outcome scores of the GOSE (cf. Figure 5). Previously, it was observed that EEG abnormalities correlate positively to complaints after mTBI [17]. GOSE scores are widely used across the TBI severity spectrum as outcome measures for recovery prediction [46, 47], where EEG mean amplitude and relative alpha power concurred to outcome prediction the most. As expected, HISC and GOSE scores correlate with each other (cf. section S6 of the Supporting Information), with a good GOSE outcome corresponding to fewer complaints in the HISC. It is possible that despite a good representation of the status of complaints after mTBI, HISC and GOSE scores are not able to fully express neurophysiological changes related to mild brain injury and to their development in time after several months. Prediction using GOSE and HISC scores as outcome measures was not possible at this stage. The lack of a relationship between neurophysiological signatures and long-term complaints may also be explained by the fact that complaints are collected several months after acute and subacute EEG measurements.

### Pathophysiological considerations

The pathophysiology involved in the various changes in neurophysiological signatures post-mTBI may, at least in part, be explained by isolated changes in synaptic transmission resulting from the trauma, as recently observed in a rodent model of mTBI [34] that may follow induced shearing stress [17]. These changes in synaptic transmission may result in EEG slowing hours to days after injury, as the EEG is essentially a readout for synaptic transmission [48]. The impairment of eye movements following mTBI has been attributed to pressure waves, damaging the inner ear and peripheral vestibular system, leading to a cascade of detrimental effects [6]. These changes are usually compensated with time, although they may also provoke a cascade of long-term detrimental effects [17, 34], resulting in persistent complaints. The lack of significant differences in RTs, fixation duration, and ERPs between the acute and subacute phases of mTBI may reflect a prolonged neuronal dysfunction, which may require additional time to recover completely.

### Limitations

Our study has limitations. First, the sample we included is not very large. Second, some of the mTBI patients were not drug-naive (cf. Table S1 of the Supporting Information), possibly affecting their task performance. Future studies with larger samples and longitudinal designs are needed to validate these results and investigate the recovery trajectory and long-term effects of mTBI on cognitive and neural functioning. Nonetheless, our findings may have important implications for the prognostication of mTBI, highlighting the importance of integrating neurophysiological features to monitor cognitive and neurophysiological functioning in mTBI patients and to verify their recovery.

## Conclusion

This study provides novel insights into the effects of mTBI on visual attention and neurophysiological functioning. We showed that in patients with mTBI, visual attention is affected. Significantly longer RTs, lower ERP mean amplitude, and slowing of EEG PDR suggest that mTBI patients have an impaired cognitive processing speed and possibly disrupted neural activity involved in stimulus evaluation. This impairment is not associated with long-term complaints.

### Supplementary Information

Below is the link to the electronic supplementary material.

Supplementary file1 (PDF 2477 KB)

## Data Availability

We are not allowed to share patients’ data.

## Code availability

The code of the visual attention task is publicly available at github.com/Valeebarons/Visual attention task. The code of data analyses for EEG and eye-tracking data is publicly available at https://github.com/Valeebarons/Analyses_eyemovements_absences
