# Supplementary material for: Neurophysiological signatures of mild traumatic brain injury in the acute and subacute phase
Source: Neurol Sci. 2024 Feb 17;45(7):3313–23. doi: 10.1007/s10072-024-07364-4 (PMC11176206; doi:10.1007/s10072-024-07364-4)
Supplement: Supplementary file 1 — Supplementary file1 (PDF 2477 KB) [file 10072_2024_7364_MOESM1_ESM.pdf]

Neurophysiological signatures of mild traumatic brain injury in the acute and subacute phase - Supplementary Information

Valentina Barone[1*], Myrthe E. de Koning[2], Harm J. van der Horn[3], Joukje van der Naalt[3], Carin Eertman-Meyer[2], Michel J.A.M. van Putten[1,2]

[1*]Clinical Neurophysiology (CNPH), TechMed Center, University of Twente, Drienerlolaan 5, Enschede, 7500 AE, the Netherlands.
[2]Department of Clinical Neurophysiology, Medisch Spectrum Twente, Koningsplein 1, Enschede, 7512 KZ, the Netherlands.
[3]Department of Neurology, University Medical Center Groningen, Hanzeplein 1, Groningen, 9713 GZ, the Netherlands.

*Corresponding author(s). E-mail(s): v.barone@utwente.nl;

S1 Task sequence description

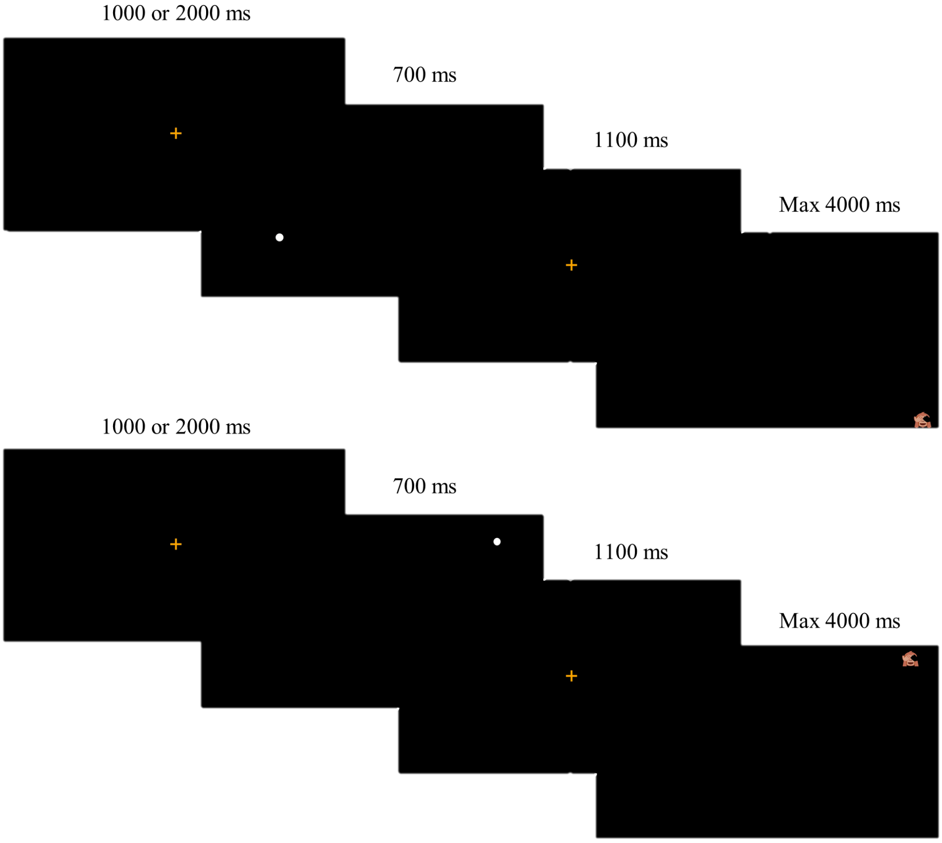

Figure S1: Sequence and timing of the visual stimuli presented during the CRT task. The task begins with a fixation cross presented at the center of the screen to establish a baseline state of attention. Next, a cue in the form of a white dot is presented, which can be either congruent (bottom figure) or incongruent (top figure) with the position of the target stimulus. Following the cue, the fixation cross is presented for 1.1 seconds. Subsequently, the target stimulus, which is a monkey's face with two hands covering both eyes, is presented either in the same position as the cue or in a different position. In this study, congruent and incongruent trials have not been differentiated and further analyzed. There are a total of eight possible positions for both the target and cue, which are presented randomly to avoid bias. If one of the monkey's hands is colored black, the subject is required to press either the left or right top button on a game controller, depending on the position of the black hand. If neither of the monkey's hands is black, no response is necessary.

S2 Demographics of patients and controls

Table S1: Demographic characteristics of patients. OM: Omeprazole; HCTZ: Hydrochlorothiazide; TAM: Tamusoline; ENL: Enalapril; DLT: Dalteparin; PNZ: Pantoprazole; ETX: Etoricoxib; MZL: Mizolastine; SAL: Salmeterol; CTZ: Cetirizine; DES: Desloratadine; AT: Atorvastatin; LIS: Lisinopril; MTX: Methotrexate; APX: Apixaban; RST: Rosuvastatin. Abnormal CT findings included minor traumatic subarachnoid hemorrhage or minor hemorrhagic concussion.

Patient #	Age	Sex	CT scan	Medication(s)
1	25	F	abnormal	none
2	75	F	normal	Clopidogrel, OM
3	18	F	normal	none
4	74	M	abnormal	HCTZ
5	59	M	normal	TAM
6	52	F	normal	ENL
7	62	M	abnormal	none
8	53	M	normal	none
9	60	F	normal	none
10	45	F	normal	none
11	45	F	normal	DLT, PNZ, ETX
12	62	F	abnormal	none
13	57	F	abnormal	none
14	52	M	normal	none
15	24	M	abnormal	none
16	22	M	abnormal	none
17	58	M	abnormal	none
18	77	M	normal	none
19	25	M	normal	none
20	19	F	abnormal	none
21	48	M	normal	MZL, SAL
22	69	F	abnormal	none
23	33	M	abnormal	none
24	18	M	normal	none
25	37	F	normal	CTZ
26	52	M	abnormal	DES
27	53	M	abnormal	AT
28	63	F	normal	LIS, MTX
29	59	M	normal	OM, APX, RST
30	34	M	normal	none
31	51	F	normal	none

Table S2: Demographic characteristics of controls.

Subject #	Age	Sex
1	61	M
2	59	M
3	63	M
4	40	F
5	27	F
6	42	M
7	60	M
8	33	F
9	26	M
10	62	M
11	42	M
12	24	F
13	65	M
14	48	M
15	24	M
16	33	M
17	32	M
18	30	M
19	34	F

S3 Reaction Times Sub-components and age

RTs are notably influenced by age differences Surwillo (1961). To test possible confounding influences given by the different mean age of our groups, we compared RTs including all participants (Fig. S2, left) to RTs after excluding patients older than 68 years old. In this way, the average age shifted from controls 42, TBI-A 46, and TBI-S 51 to controls 42, TBI-A 41, and TBI-S 46. As shown in figure S2, right, PS and VRT decreased 5-11 ms for TBI-A and TBI-S. The sample's mean difference and significance remained unaltered.

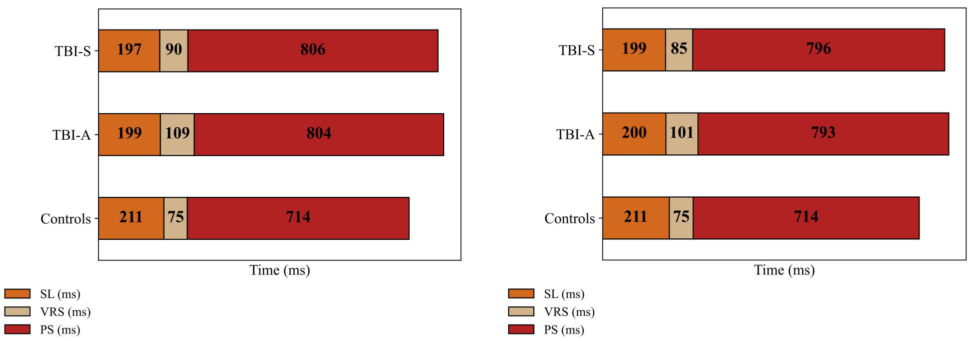

Figure S2: Left: Values of RT sub-components including all participants. In orange saccadic latency (SL), in yellow visual reaction time (VRT), and in red processing speed (PS). VRT is significantly slower for TBI-A, while PS is significantly slower for controls ($p < .05$). VRT and PS differ significantly among the three groups and between TBI patients and controls. SL does not show any statistically significant difference. Right: Values of RT sub-components after excluding patients older than 68 years old. In orange saccadic latency (SL), in yellow visual reaction time (VRT), and in red processing speed (PS). Values of PS and VRT decreased for TBI-A and TBI-S (5-11 ms). All the statistical differences found for the left panel are consistent.

S4 ERPs and age

Age can affect ERPs mean amplitude and peak latency van Dinteren et al (2014). To test the possible influence of age on our ERP results we determined ERP mean amplitude and peak latency for channel Pz, P3, and P4 excluding patients older than 68, to obtain a closer average age across groups (controls 42, TBI-A 41, and TBI-S 46). In Fig. S3 ERP results are shown. As for the results reported in our manuscript, the mean amplitude is significantly bigger for controls compared to TBI patients (mean difference 3.2μV vs 3.4μV including all patients), while the peak latency does not statistically differ among groups.

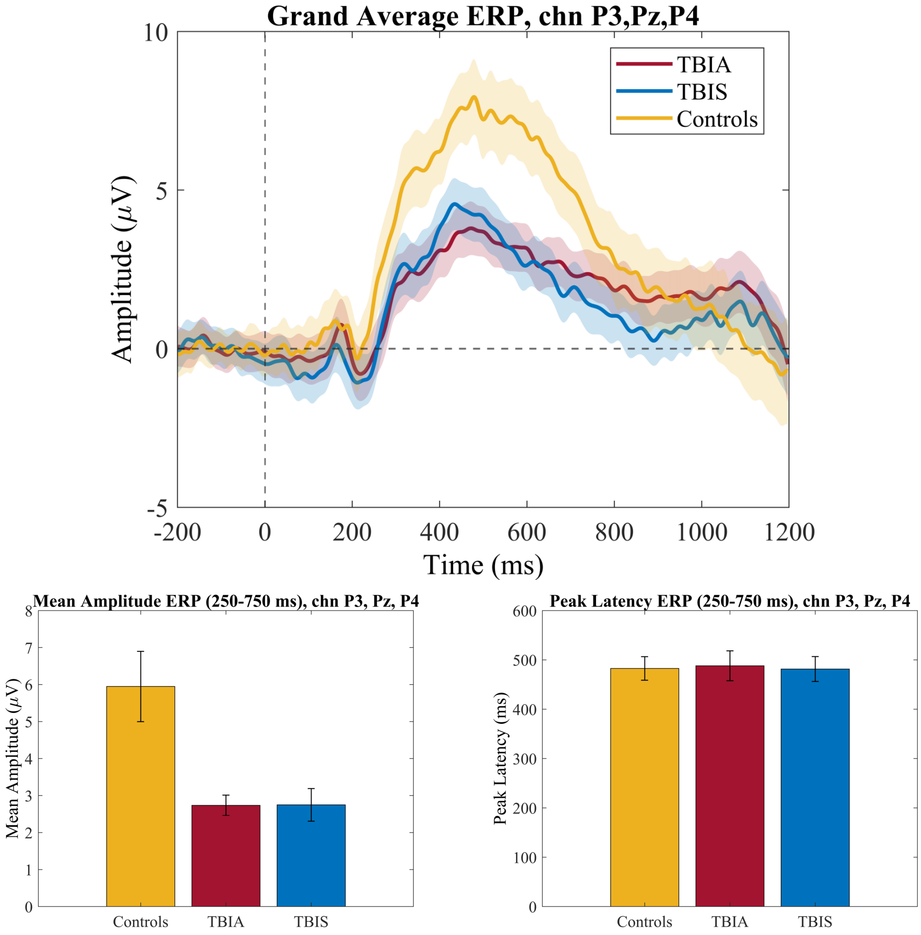

Figure S3: Top: Grand Average ERP for channel P3, Pz, P4 of the three groups without patients older than 68. The colored area around the averaged signal represents the standard error of the mean for each time point. Bottom left: mean amplitude between 250-750 ms is significantly different across groups. The mean difference (MD) between controls and both TBI groups is 3.2µV. No significant difference is found between TBI-A and TBI-S. Bottom right: Peak latency between 250-750 ms. No significant difference across groups is found (MD of controls vs TBI-A= 5 ms; MD of controls vs TBI-S= 1, MD of TBI-A vs TBI-S= 7 ms).

S5 Outcome measures - HISC

HISC scores range between 0 and 17. We assessed possible associations of HISC scores with total RT and peak frequency in the alpha band during EC EEG. In Fig. S4, weak or no associations between the complaints of TBI patients and our

neurophysiological variables can be seen. No statistically significant correlation was found.

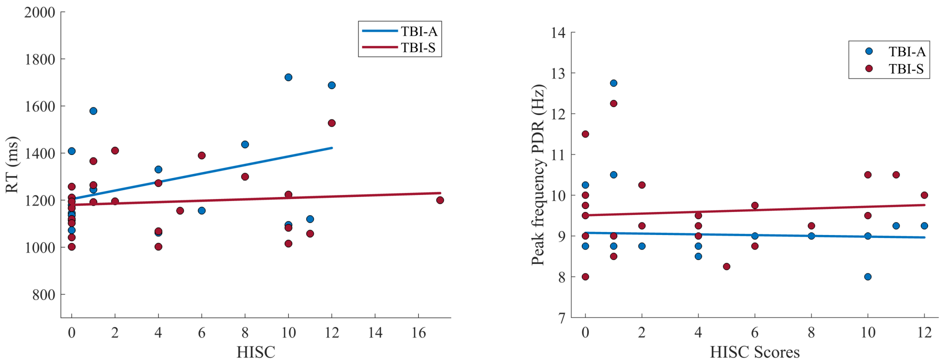

Figure S4: Left: Association between HISC outcomes and total RT for TBI-A (blue) and TBI-S (red). A positive trend is clearly visible for TBI-A patients, but no significant correlation was found. Right: Association between HISC outcomes and peak frequency in the alpha band during resting EEG with EC for TBI-A (blue) and TBI-S (red). No significant association could be found.

S6 Association between HISC and GOSE

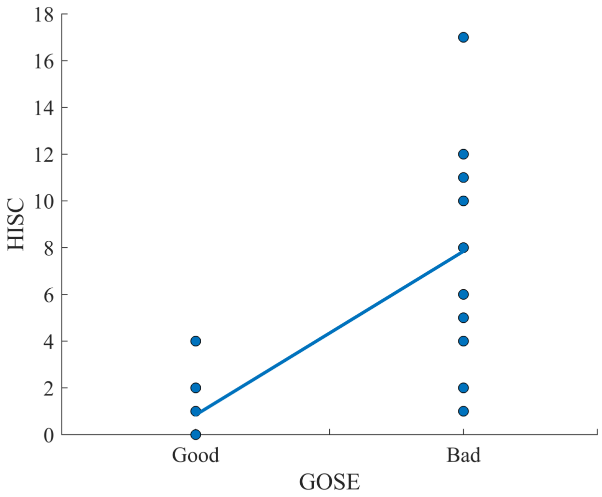

Figure S5: Correlation between HISC and GOSE scores. A significant, positive correlation is found between HISC and GOSE scores: a worse outcome of the GOSE corresponds to more complaints after concussion with the HISC.

S7 Follow-up patients

EEG mean amplitude can vary extensively between subjects Morioka et al (2015). To test the possible influence of subject-related EEG variability we performed our analysis on resting EEG and ERPs on the patients who performed both measurements, i.e., N= 25 for resting EEG, N= 14 for ERPs. In Fig. S6, the PDR of all the groups, both at EC and EO, appear slightly higher with follow-up patients only. Differences in peak frequency (Fig. S6, right) do not differ from our result presented in Fig. 2 of the manuscript. In Fig. S7, ERP results with 14 patients with both measurements do no differ from our results with all patients included.

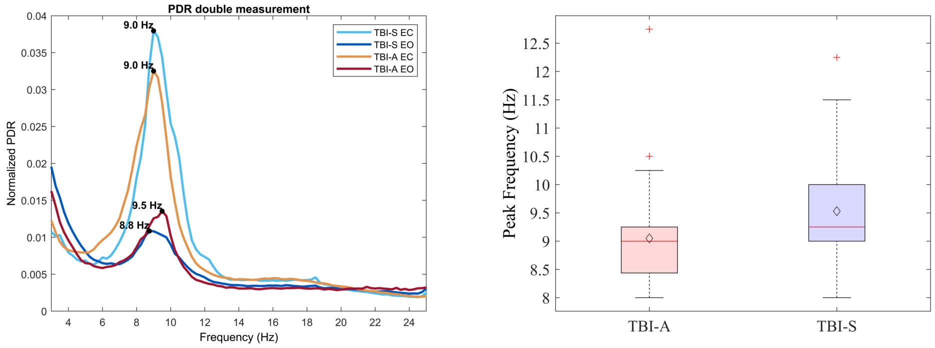

Figure S6: Left: Normalized PDR of eye closed (EC) and eyes open (EO) during resting EEG over frequency. Patients of TBI-A and TBI-S are the same (N= 25). The peak frequency in the alpha band is shown at the peak of each curve. Right: Peak frequency of parieto-occipital channels in the alpha band during resting EEG with eyes closed of patients with a double measurement only (N= 25). A mean difference of 0.5 Hz is found between TBI-A and TBI-S ($p < .05$).

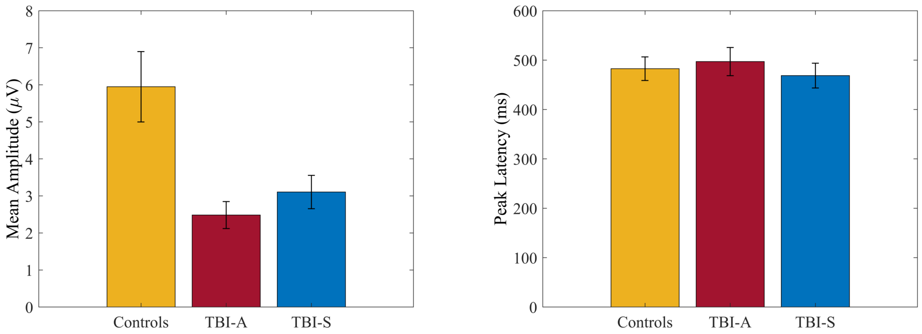

Figure S7: Left: ERP mean amplitude between 250-750 ms between patients with double measurement (N= 14). Mean difference is significantly different across groups. The mean difference (MD) between controls and TBI-A is 3.5μV, MD between controls and TBI-S is 2.8μV. No significant difference is found between TBI-A and TBI-S. Right: ERP peak latency between 250-750 ms. No significant difference across groups is found (MD of controls vs TBI-A= 14 ms; MD of controls vs TBI-S= 14, MD of TBI-A vs TBI-S = 28 ms).

S8 Non-parametric statistics for ET and ERP variables

We determined the normality of the dependent variables of our study. Visually, through an inspection using histograms and Q-Q plots, our data appear normally distributed. Nevertheless, only two of our variables (ERP mean amplitude and ET

fixation duration) passed the Shapiro-Wilk test ($p > 0.05$). These contrasting results are possibly due to the small sample size we used. The analyses of ERP and ET variables reported in our manuscript use parametric statistics, here we want to prove the robustness of our results by applying non-parametric statistical tests to ERP and ET variables that do not pass the Shapiro-Wilk test, i.e., peak latency ERP, RT, SL, VRT, PS. First, we applied the Kruskal-Wallis test to determine the significance of the fixed effects of our variables. Thereafter, to evaluate pairwise differences, we used Dunn's test.

Results are shown in Table S3.

Table S3: Results of non-parametric statics. D-test: Dunn's test; HC: healthy controls. PL: peak latency of ERP variables.

Variable #	Kruskal-Wallis	D-test HC vs TBI-A	D-test HC vs TBI-S	D-test TBI-A vs TBI-S
PL	0.8	0.45	0.45	0.8
RT	0.005**	0.006**	0.004**	0.4
SL	0.4	0.3	0.2	0.4
VRT	0.002**	0.0008**	0.02*	0.06
PS	0.03*	0.02*	0.02*	0.5

* $p < 0.05$
** $p < 0.01$

The results shown by non-parametric statistics are in line with the ANOVA and pairwise results reported in our manuscript, except for VRT, which here shows significant differences between controls and TBI-S too, while in the manuscript this is not the case.

S9 Normality check of dependent variables

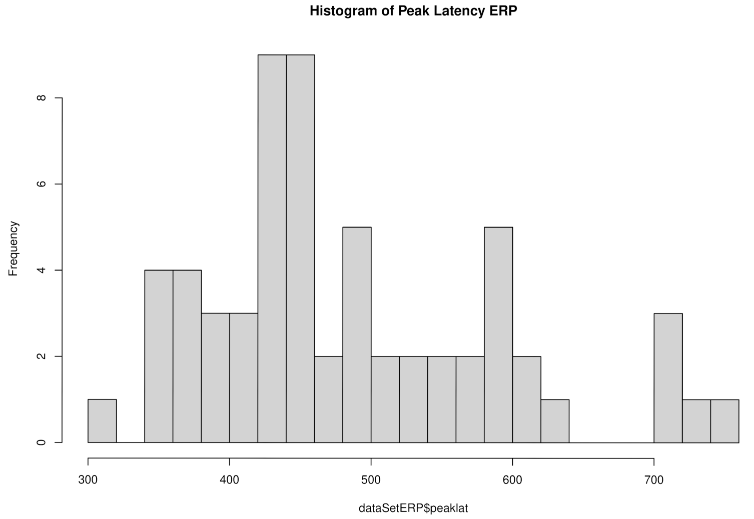

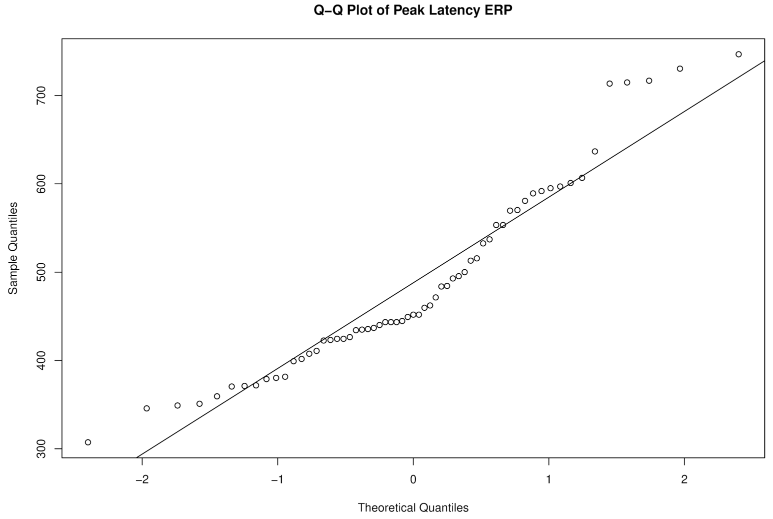

Figure S8: Histogram (left) and Q-Q normality plot (right) to check the distribution of the dependent variable peak latency ERP. Despite the data did not pass the Shapiro-Wilk test, the histogram and Q-Q normality plot here reported show an overall normal distribution.

S10 Normality check of residuals

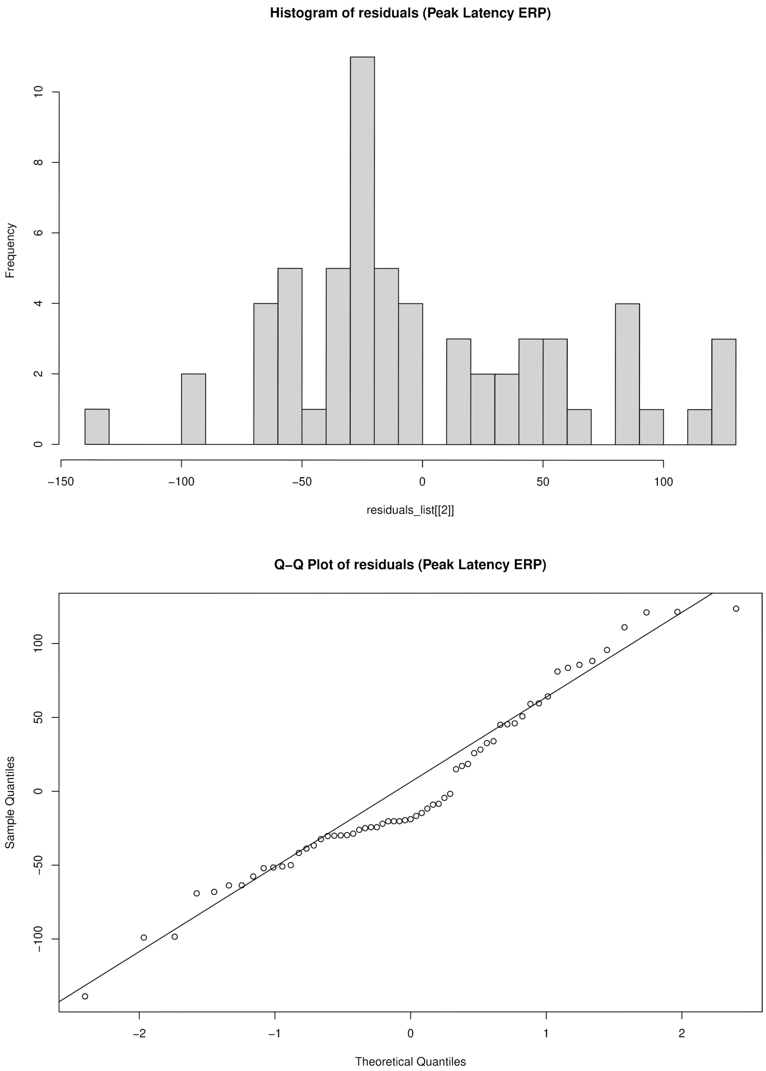

Figure S9: Histogram (left) and Q-Q normality plot (right) to check the distribution of the residuals of the linear mixed effect model for the variable peak latency ERP. Despite the data did not pass the Shapiro-Wilk test, the histogram and Q-Q normality plot here show an overall normal distribution.

References

van Dinteren R, Arns M, Jongsma MLA, et al (2014) P300 Development across the Lifespan: A SystematicReview and Meta-Analysis. Plos ONE 9(2):1–13

Morioka H, Kanemura A, Hirayama J, et al (2015) Learning a common dictionary for subject-transfer decoding with resting calibration. NeuroImage 111:167–178

Surwillo WW (1961) Frequency of the ’Alpha’ Rhythm, Reaction Time and Age. Nature 191:823–824
